# 1-Benzyl-4′,5′-diphenyl­piperidine-3-spiro-3′-pyrrolidine-2′-spiro-3′′-indoline-4,2′′-dione

**DOI:** 10.1107/S160053680804141X

**Published:** 2008-12-17

**Authors:** J. Suresh, R. Suresh Kumar, A. Rajapriya, S. Perumal, P. L. Nilantha Lakshman

**Affiliations:** a Department of Physics, The Madura College, Madurai 625011, India; b School of Chemistry, Madurai Kamaraj University, Madurai 625021, India; c Department of Food Science and Technology, Faculty of Agriculture, University of Ruhuna, Mapalana, Kamburupitiya 81100, Sri Lanka

## Abstract

The asymmetric unit of the title compound, C_34_H_31_N_3_O_2_, consists of two independent mol­ecules which differ slightly in the orientations of the phenyl rings with respect to the pyrrolidine ring. In both mol­ecules, the piperidin-4-one ring adopts a chair conformation, whereas the pyrrolidine ring adopts an envelope conformation in one of the mol­ecules and a twisted conformation in the other. An intra­molecular C—H⋯O hydrogen bond is observed. The crystal packing is stabilized by inter­molecular N—H⋯O hydrogen bonds and C—H⋯π inter­actions.

## Related literature

For the biological activities of oxindole derivatives, see: Bhattacharya *et al.* (1982 ▶); Glover *et al.* (1998 ▶); Govind *et al.* (2004 ▶); Hilton *et al.* (2000 ▶); Jeyabharathi *et al.* (2001 ▶); Kirsch *et al.* (2004 ▶); Klumpp *et al.* (1998 ▶); Kumar *et al.* (1993 ▶, 2006 ▶); Medvedev *et al.* (1996 ▶). For bond-length data, see: Allen *et al.* (1987 ▶).
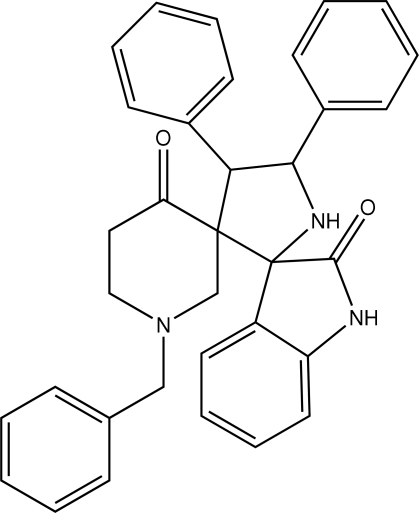


## Experimental

### 

#### Crystal data

C_34_H_31_N_3_O_2_
*M* *_r_* = 513.62
Triclinic, 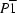

*a* = 10.8575 (3) Å
*b* = 13.7909 (5) Å
*c* = 20.5053 (9) Å
α = 89.767 (6)°
β = 75.056 (4)°
γ = 71.846 (3)°
*V* = 2809.1 (2) Å^3^
*Z* = 4
Mo *K*α radiation
μ = 0.08 mm^−1^
*T* = 293 (2) K
0.17 × 0.13 × 0.11 mm

#### Data collection

Nonius MACH-3 diffractometer
Absorption correction: ψ scan (North *et al.*, 1968 ▶) *T*_min_ = 0.988, *T*_max_ = 0.992
11592 measured reflections
9879 independent reflections
4342 reflections with *I* > 2σ(*I*)
*R*_int_ = 0.026
2 standard reflections frequency: 60 min intensity decay: none

#### Refinement

*R*[*F* ^2^ > 2σ(*F* ^2^)] = 0.053
*wR*(*F* ^2^) = 0.157
*S* = 0.99
9879 reflections
711 parameters
2 restraints
H atoms treated by a mixture of independent and constrained refinement
Δρ_max_ = 0.26 e Å^−3^
Δρ_min_ = −0.28 e Å^−3^

### 

Data collection: *CAD-4 EXPRESS* (Enraf-Nonius, 1994 ▶); cell refinement: *CAD-4 EXPRESS*; data reduction: *XCAD4* (Harms & Wocadlo, 1996 ▶); program(s) used to solve structure: *SHELXS97* (Sheldrick, 2008 ▶); program(s) used to refine structure: *SHELXL97* (Sheldrick, 2008 ▶); molecular graphics: *PLATON* (Spek, 2003 ▶); software used to prepare material for publication: *SHELXL97*.

## Supplementary Material

Crystal structure: contains datablocks global, I. DOI: 10.1107/S160053680804141X/ci2737sup1.cif

Structure factors: contains datablocks I. DOI: 10.1107/S160053680804141X/ci2737Isup2.hkl

Additional supplementary materials:  crystallographic information; 3D view; checkCIF report

## Figures and Tables

**Table 1:** Hydrogen-bond geometry (Å, °)

*D*—H⋯*A*	*D*—H	H⋯*A*	*D*⋯*A*	*D*—H⋯*A*
N3—H3⋯O4	0.86	2.03	2.881 (3)	169
N6—H6⋯O2	0.86	2.05	2.896 (3)	170
C40—H40*A*⋯O4	0.97	2.35	2.925 (4)	118
C12—H12⋯*Cg*1^i^	0.93	2.80	3.586 (6)	144

Symmetry code: (i) 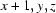. *Cg*1 is the centroid of the C29–C34 ring.

**Fig. 1.**
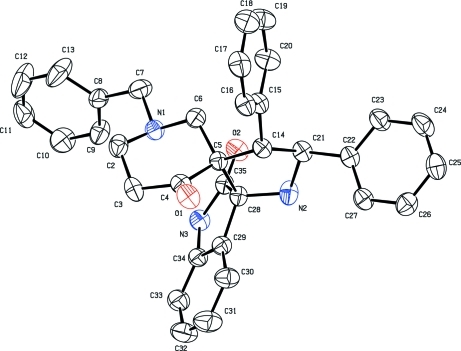
Molecule-1 of the two molecules in the asymmetric unit, showing the atom-numbering scheme. Displacement ellipsoids are drawn at the 30% probability level. H-atoms have been omitted for clarity.

**Fig. 2.**
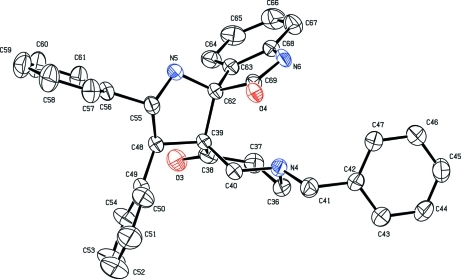
Molecule-2 of the two molecules in the asymmetric unit, showing the atom-numbering scheme. Displacement ellipsoids are drawn at the 30% probability level. H-atoms have been omitted for clarity.

**Fig. 3.**
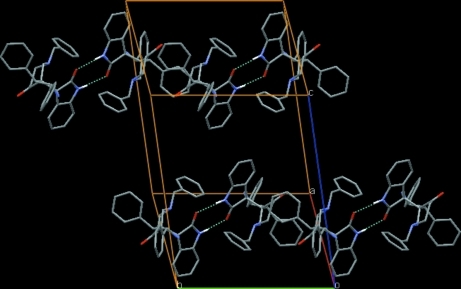
A partial packing view showing the intermolecular N—H···O interactions (dashed lines) generating an R22(8) graph-set motif in the title compound. H atoms are omitted for clarity.

## supplementary crystallographic information

### Comment

The development of new efficient methods to synthesize N-heterocycles with structural diversity is one of the major interests of modern synthetic organic chemists (Kirsch *et al.*, 2004). Oxindole derivatives are known to possess a variety of biological activities (Klumpp *et al.*, 1998) such as (i) a potent inhibitor of monoamine oxidase (MAO) in human urine and rat tissues (Glover *et al.*, 1998) (ii) inhibition of several enzymes such as acetylcholinestrease (AChE) (Kumar *et al.*, 1993) and atrial natriuretic peptide-stimulated guanylate cyclase and (iii) a potent antagonist of *in vitro* receptor binding by atrial natriuretic peptide (Medvedev *et al.*, 1996) besides possessing a wide range of central nervous system activities (Bhattacharya *et al.*, 1982). The derivatives of spirooxindole ring systems are used as antimicrobial, antitumour agents and as inhibitors of the human NKI receptor besides being found in a number of alkaloids like horsifiline, spirotryprostatin and (+)elacomine (Hilton *et al.*, 2000). Our interest in preparing pharmacologically active pyrrolidines led us to the title compound, and we have undertaken X-ray crystal structure determination of these compounds in order to establish their conformations.

The asymmetric unit of the title compound contains two independent molecules, and these pair has almost identical geometry (Fig. 1 and Fig. 2). In both the molecules, the bond lengths and bond angles show normal values and agree with each other (Allen *et al.*, 1987). The sums of the angles at atoms N2 and N5 of the pyrrolidine rings 342.3 and 333.4°, respectively, are in accordance with *sp*^3^-hybridization, and sums of the angles at atoms N3 and N6 of the indolin-2-one moiety 360 and 359.8° confirms the *sp*^2^-hybridization (Govind *et al.*, 2004; Kumar *et al.*, 2006; Jeyabharathi *et al.*, 2001). The bond lengths within the indolin-2-one moiety match with those in similar structures (Kumar *et al.*, 2006; Jeyabharathi *et al.*, 2001).

In one of the independent molecules the pyrrolidine ring (in ring N5/C55/C48/C39/C62, C55 is the flap atom) adopts an envelope conformation and in the other it exhibits a twisted conformation. In the indolin-2-one ring systems, the benzene and pyrrole rings are individually planar and make dihedral angles of 3.8 (2) and 1.7 (2)°, while atoms O2 and O4 deviate from the pyrrole ring of the indolin-2-one system by -0.224 (5) and 0.117 (5) Å, respectively, because of the different interactions in which these O atoms are involved (Table 1). The orientations of the phenyl groups with respect to the pyrrolidine ring differ slightly in the two independent molecules.

N—H···O hydrogen bonds between the two molecules in the asymmetric unit generate an *R*_2_^2^(8) graph set motif (Table 1 and Fig. 3). In addition, a C—H···π interaction (Table 1) is also found.

### Experimental

A mixture of 1-benzyl-4-piperidinone (0.2 g, 0.001 mol), isatin (0.156 g, 0.001 mol) and phenylglycine (0.320 g, 0.002 mol) in methanol-water (2:1, 30 ml) were refluxed in a water bath for 24 h. After completion of the reaction as monitored by TLC, the excess solvent was removed under vacuum and the residue subjected to flash column chromatography using petroleum ether-ethyl acetate mixture (8:2 *v*/*v*) as eluent (yield: 0.220 g, 41%; m.p. 464–465 K)

### Refinement

H atoms attached to N2 and N5 are located in a difference map and refined with an N—H distance restraint of 0.82 (2)Å.  All other H atoms were placed at calculated positions and allowed to ride on their carrier atoms with C—H = 0.93–0.98 Å and N—H = 0.86 Å. *U*_iso_= 1.2*U*_eq_(C,N) for CH_2_, CH and NH groups.

### Crystal data

**Table:**

C_34_H_31_N_3_O_2_	*Z* = 4
*M**_r_* = 513.62	*F*(000) = 1088
Triclinic, *P*1	*D*_x_ = 1.214 Mg m^−^^3^
Hall symbol: -P 1	Mo *K*α radiation, λ = 0.71073 Å
*a* = 10.8575 (3) Å	Cell parameters from 25 reflections
*b* = 13.7909 (5) Å	θ = 2–25°
*c* = 20.5053 (9) Å	µ = 0.08 mm^−^^1^
α = 89.767 (6)°	*T* = 293 K
β = 75.056 (4)°	Block, colourless
γ = 71.846 (3)°	0.17 × 0.13 × 0.11 mm
*V* = 2809.1 (2) Å^3^	

### Data collection

**Table:**

Nonius MACH-3 diffractometer	4342 reflections with *I* > 2σ(*I*)
Radiation source: fine-focus sealed tube	*R*_int_ = 0.026
graphite	θ_max_ = 25.0°, θ_min_ = 2.0°
ω–2θ scans	*h* = −1→12
Absorption correction: ψ scan (North *et al.*, 1968)	*k* = −15→16
*T*_min_ = 0.988, *T*_max_ = 0.992	*l* = −23→24
11592 measured reflections	2 standard reflections every 60 min
9879 independent reflections	intensity decay: none

### Refinement

**Table:**

Refinement on *F*^2^	Primary atom site location: structure-invariant direct methods
Least-squares matrix: full	Secondary atom site location: difference Fourier map
*R*[*F*^2^ > 2σ(*F*^2^)] = 0.053	Hydrogen site location: inferred from neighbouring sites
*wR*(*F*^2^) = 0.157	H atoms treated by a mixture of independent and constrained refinement
*S* = 0.99	*w* = 1/[σ^2^(*F*_o_^2^) + (0.0638*P*)^2^ + 0.294*P*] where *P* = (*F*_o_^2^ + 2*F*_c_^2^)/3
9879 reflections	(Δ/σ)_max_ = 0.001
711 parameters	Δρ_max_ = 0.26 e Å^−^^3^
2 restraints	Δρ_min_ = −0.28 e Å^−^^3^

### Special details

**Table:**

Geometry. All e.s.d.'s (except the e.s.d. in the dihedral angle between two l.s. planes) are estimated using the full covariance matrix. The cell e.s.d.'s are taken into account individually in the estimation of e.s.d.'s in distances, angles and torsion angles; correlations between e.s.d.'s in cell parameters are only used when they are defined by crystal symmetry. An approximate (isotropic) treatment of cell e.s.d.'s is used for estimating e.s.d.'s involving l.s. planes.
Refinement. Refinement of *F*^2^ against ALL reflections. The weighted *R*-factor *wR* and goodness of fit *S* are based on *F*^2^, conventional *R*-factors *R* are based on *F*, with *F* set to zero for negative *F*^2^. The threshold expression of *F*^2^ > σ(*F*^2^) is used only for calculating *R*-factors(gt) *etc*. and is not relevant to the choice of reflections for refinement. *R*-factors based on *F*^2^ are statistically about twice as large as those based on *F*, and *R*- factors based on ALL data will be even larger.

### Fractional atomic coordinates and isotropic or equivalent isotropic displacement parameters (Å^2^)

**Table:**

	*x*	*y*	*z*	*U*_iso_*/*U*_eq_	
C2	0.3213 (3)	1.0209 (3)	0.22160 (17)	0.0653 (10)	
H2A	0.4042	0.9839	0.1881	0.078*	
H2B	0.3361	1.0771	0.2432	0.078*	
C3	0.2105 (4)	1.0628 (3)	0.18704 (16)	0.0649 (10)	
H3A	0.2301	1.1155	0.1587	0.078*	
H3B	0.2089	1.0082	0.1579	0.078*	
C4	0.0747 (4)	1.1068 (3)	0.23641 (16)	0.0539 (9)	
C5	0.0408 (3)	1.0445 (2)	0.29616 (14)	0.0462 (8)	
C6	0.1645 (3)	1.0109 (2)	0.32383 (16)	0.0548 (9)	
H6A	0.1783	1.0709	0.3412	0.066*	
H6B	0.1485	0.9689	0.3611	0.066*	
C7	0.3934 (3)	0.9066 (3)	0.30452 (18)	0.0737 (11)	
H7A	0.3632	0.8643	0.3389	0.088*	
H7B	0.4111	0.9606	0.3270	0.088*	
C8	0.5221 (3)	0.8422 (3)	0.25547 (16)	0.0599 (9)	
C9	0.5243 (4)	0.7615 (3)	0.21722 (19)	0.0713 (10)	
H9	0.4447	0.7471	0.2207	0.086*	
C10	0.6414 (5)	0.7006 (3)	0.1736 (2)	0.0855 (12)	
H10	0.6403	0.6460	0.1476	0.103*	
C11	0.7578 (4)	0.7192 (4)	0.1682 (2)	0.0893 (14)	
H11	0.8376	0.6775	0.1389	0.107*	
C12	0.7569 (5)	0.7985 (4)	0.2055 (3)	0.130 (2)	
H12	0.8371	0.8118	0.2021	0.156*	
C13	0.6410 (4)	0.8604 (4)	0.2487 (3)	0.1121 (17)	
H13	0.6430	0.9156	0.2737	0.135*	
C14	−0.0888 (3)	1.1077 (2)	0.35148 (14)	0.0497 (8)	
H14	−0.1512	1.1495	0.3275	0.060*	
C15	−0.0687 (3)	1.1807 (2)	0.39981 (16)	0.0535 (8)	
C16	−0.0916 (3)	1.2825 (2)	0.38747 (17)	0.0616 (9)	
H16	−0.1238	1.3060	0.3505	0.074*	
C17	−0.0682 (4)	1.3501 (3)	0.4284 (2)	0.0759 (11)	
H17	−0.0845	1.4182	0.4187	0.091*	
C18	−0.0207 (4)	1.3173 (3)	0.4833 (2)	0.0865 (13)	
H18	−0.0026	1.3624	0.5102	0.104*	
C19	−0.0005 (4)	1.2176 (3)	0.49794 (19)	0.0861 (13)	
H19	0.0296	1.1951	0.5356	0.103*	
C20	−0.0247 (4)	1.1505 (3)	0.45711 (18)	0.0716 (11)	
H20	−0.0112	1.0832	0.4681	0.086*	
C21	−0.1493 (3)	1.0263 (2)	0.38361 (15)	0.0513 (8)	
H21	−0.0978	0.9889	0.4139	0.062*	
C22	−0.2973 (3)	1.0681 (2)	0.42099 (16)	0.0514 (8)	
C23	−0.3386 (4)	1.0833 (3)	0.49076 (17)	0.0702 (10)	
H23	−0.2748	1.0666	0.5152	0.084*	
C24	−0.4738 (4)	1.1231 (3)	0.52476 (19)	0.0836 (12)	
H24	−0.5001	1.1330	0.5718	0.100*	
C25	−0.5676 (4)	1.1476 (3)	0.4899 (2)	0.0757 (11)	
H25	−0.6585	1.1738	0.5129	0.091*	
C26	−0.5290 (4)	1.1337 (3)	0.42030 (19)	0.0681 (10)	
H26	−0.5935	1.1508	0.3962	0.082*	
C27	−0.3937 (4)	1.0941 (2)	0.38614 (17)	0.0590 (9)	
H27	−0.3679	1.0850	0.3391	0.071*	
C28	0.0013 (3)	0.9504 (2)	0.27379 (15)	0.0465 (8)	
C29	−0.0114 (3)	0.9474 (2)	0.20229 (15)	0.0515 (8)	
C30	−0.0968 (4)	1.0131 (3)	0.17111 (19)	0.0706 (10)	
H30	−0.1616	1.0721	0.1949	0.085*	
C31	−0.0858 (5)	0.9907 (3)	0.1039 (2)	0.0920 (13)	
H31	−0.1425	1.0355	0.0821	0.110*	
C32	0.0089 (5)	0.9023 (4)	0.0690 (2)	0.0883 (13)	
H32	0.0144	0.8881	0.0239	0.106*	
C33	0.0958 (4)	0.8342 (3)	0.09954 (18)	0.0693 (10)	
H33	0.1600	0.7749	0.0759	0.083*	
C34	0.0831 (3)	0.8583 (2)	0.16613 (16)	0.0521 (8)	
C35	0.1075 (3)	0.8443 (2)	0.27324 (17)	0.0473 (8)	
C36	0.1749 (3)	0.3551 (3)	0.26427 (17)	0.0602 (9)	
H36A	0.1644	0.3061	0.2340	0.072*	
H36B	0.0918	0.3806	0.3001	0.072*	
C37	0.2893 (3)	0.3029 (2)	0.29445 (17)	0.0611 (9)	
H37A	0.2865	0.3475	0.3316	0.073*	
H37B	0.2768	0.2406	0.3128	0.073*	
C38	0.4256 (4)	0.2763 (3)	0.24421 (16)	0.0557 (9)	
C39	0.4477 (3)	0.3585 (2)	0.19573 (14)	0.0443 (8)	
C40	0.3214 (3)	0.3985 (2)	0.17048 (14)	0.0502 (8)	
H40A	0.3305	0.4517	0.1403	0.060*	
H40B	0.3120	0.3432	0.1451	0.060*	
C41	0.0853 (3)	0.4983 (3)	0.20349 (16)	0.0645 (10)	
H41A	0.0652	0.4534	0.1744	0.077*	
H41B	0.1077	0.5518	0.1769	0.077*	
C42	−0.0370 (3)	0.5458 (3)	0.26163 (17)	0.0575 (9)	
C43	−0.1500 (4)	0.5157 (3)	0.27214 (19)	0.0707 (10)	
H43	−0.1514	0.4651	0.2427	0.085*	
C44	−0.2616 (4)	0.5601 (3)	0.3262 (2)	0.0798 (11)	
H44	−0.3374	0.5395	0.3325	0.096*	
C45	−0.2611 (4)	0.6334 (3)	0.3699 (2)	0.0814 (12)	
H45	−0.3365	0.6636	0.4058	0.098*	
C46	−0.1486 (4)	0.6625 (3)	0.3608 (2)	0.0901 (13)	
H46	−0.1469	0.7116	0.3912	0.108*	
C47	−0.0377 (4)	0.6192 (3)	0.3067 (2)	0.0794 (11)	
H47	0.0377	0.6402	0.3007	0.095*	
C48	0.5777 (3)	0.3160 (2)	0.13665 (15)	0.0506 (8)	
H48	0.6418	0.2631	0.1544	0.061*	
C49	0.5631 (3)	0.2665 (3)	0.07503 (19)	0.0653 (10)	
C50	0.5388 (4)	0.3202 (3)	0.01969 (19)	0.0852 (12)	
H50	0.5311	0.3893	0.0203	0.102*	
C51	0.5260 (5)	0.2725 (5)	−0.0363 (2)	0.1161 (18)	
H51	0.5105	0.3094	−0.0731	0.139*	
C52	0.5360 (7)	0.1722 (6)	−0.0375 (3)	0.144 (3)	
H52	0.5248	0.1410	−0.0747	0.173*	
C53	0.5628 (6)	0.1159 (5)	0.0158 (3)	0.135 (2)	
H53	0.5715	0.0466	0.0142	0.162*	
C54	0.5769 (4)	0.1638 (3)	0.0729 (2)	0.0968 (14)	
H54	0.5955	0.1262	0.1089	0.116*	
C55	0.6326 (3)	0.4065 (2)	0.12493 (14)	0.0508 (8)	
H55	0.5766	0.4589	0.1029	0.061*	
C56	0.7779 (3)	0.3798 (2)	0.08585 (16)	0.0529 (8)	
C57	0.8154 (4)	0.4215 (3)	0.02578 (18)	0.0745 (11)	
H57	0.7497	0.4671	0.0092	0.089*	
C58	0.9484 (4)	0.3968 (4)	−0.0100 (2)	0.0937 (14)	
H58	0.9721	0.4253	−0.0507	0.112*	
C59	1.0461 (4)	0.3303 (4)	0.0142 (2)	0.0895 (13)	
H59	1.1360	0.3132	−0.0102	0.107*	
C60	1.0116 (4)	0.2895 (3)	0.0735 (2)	0.0828 (12)	
H60	1.0779	0.2450	0.0903	0.099*	
C61	0.8786 (4)	0.3141 (3)	0.10893 (18)	0.0688 (10)	
H61	0.8561	0.2854	0.1497	0.083*	
C62	0.4766 (3)	0.4471 (2)	0.23407 (14)	0.0442 (8)	
C63	0.4689 (3)	0.4410 (2)	0.30844 (15)	0.0471 (8)	
C64	0.5400 (3)	0.3672 (2)	0.34215 (17)	0.0607 (9)	
H64	0.6061	0.3088	0.3183	0.073*	
C65	0.5122 (4)	0.3809 (3)	0.41216 (19)	0.0748 (11)	
H65	0.5581	0.3309	0.4357	0.090*	
C66	0.4165 (4)	0.4689 (4)	0.44632 (18)	0.0811 (12)	
H66	0.3974	0.4769	0.4932	0.097*	
C67	0.3482 (4)	0.5453 (3)	0.41317 (17)	0.0686 (10)	
H67	0.2852	0.6052	0.4368	0.082*	
C68	0.3759 (3)	0.5302 (2)	0.34428 (15)	0.0497 (8)	
C69	0.3734 (3)	0.5552 (2)	0.23508 (16)	0.0455 (8)	
N1	0.2856 (3)	0.9529 (2)	0.27199 (13)	0.0554 (7)	
N2	−0.1264 (3)	0.9597 (2)	0.32336 (15)	0.0609 (8)	
N3	0.1525 (3)	0.79994 (18)	0.20924 (13)	0.0540 (7)	
H3	0.2162	0.7426	0.1965	0.065*	
N4	0.2014 (2)	0.43952 (18)	0.22703 (12)	0.0494 (7)	
N5	0.6116 (3)	0.4436 (2)	0.19536 (13)	0.0520 (7)	
N6	0.3222 (2)	0.59638 (18)	0.29943 (12)	0.0523 (7)	
H6	0.2636	0.6561	0.3116	0.063*	
O1	−0.0030 (2)	1.18932 (17)	0.23002 (11)	0.0696 (7)	
O2	0.1366 (2)	0.80371 (15)	0.32311 (11)	0.0597 (6)	
O3	0.5124 (2)	0.19456 (17)	0.24193 (12)	0.0717 (7)	
O4	0.3524 (2)	0.59973 (14)	0.18487 (10)	0.0541 (6)	
H2	−0.144 (3)	0.9069 (16)	0.3322 (13)	0.045 (10)*	
H5	0.628 (4)	0.499 (2)	0.197 (2)	0.111 (16)*	

### Atomic displacement parameters (Å^2^)

**Table:**

	*U*^11^	*U*^22^	*U*^33^	*U*^12^	*U*^13^	*U*^23^
C2	0.056 (2)	0.062 (2)	0.069 (2)	−0.0212 (19)	0.000 (2)	−0.0134 (19)
C3	0.074 (3)	0.057 (2)	0.053 (2)	−0.023 (2)	0.004 (2)	0.0023 (17)
C4	0.064 (2)	0.0409 (19)	0.054 (2)	−0.0159 (18)	−0.0130 (18)	−0.0044 (16)
C5	0.048 (2)	0.0389 (17)	0.0461 (18)	−0.0118 (15)	−0.0065 (15)	−0.0037 (14)
C6	0.052 (2)	0.0500 (19)	0.058 (2)	−0.0133 (17)	−0.0096 (17)	−0.0114 (16)
C7	0.056 (2)	0.087 (3)	0.073 (2)	−0.011 (2)	−0.024 (2)	−0.018 (2)
C8	0.045 (2)	0.071 (2)	0.059 (2)	−0.0127 (18)	−0.0118 (17)	−0.0086 (18)
C9	0.056 (2)	0.066 (2)	0.091 (3)	−0.025 (2)	−0.009 (2)	−0.006 (2)
C10	0.077 (3)	0.071 (3)	0.093 (3)	−0.010 (2)	−0.016 (3)	−0.020 (2)
C11	0.057 (3)	0.102 (4)	0.070 (3)	0.007 (3)	0.009 (2)	0.001 (2)
C12	0.051 (3)	0.137 (5)	0.185 (6)	−0.036 (3)	0.006 (3)	−0.032 (4)
C13	0.063 (3)	0.107 (4)	0.167 (5)	−0.036 (3)	−0.020 (3)	−0.052 (3)
C14	0.050 (2)	0.0394 (17)	0.0494 (18)	−0.0040 (15)	−0.0087 (16)	−0.0015 (15)
C15	0.054 (2)	0.0416 (19)	0.053 (2)	−0.0054 (16)	−0.0059 (17)	−0.0056 (16)
C16	0.069 (2)	0.046 (2)	0.058 (2)	−0.0125 (18)	−0.0034 (18)	−0.0045 (17)
C17	0.088 (3)	0.051 (2)	0.076 (3)	−0.023 (2)	0.002 (2)	−0.015 (2)
C18	0.093 (3)	0.081 (3)	0.077 (3)	−0.026 (3)	−0.010 (3)	−0.029 (2)
C19	0.104 (3)	0.077 (3)	0.069 (3)	−0.014 (3)	−0.029 (2)	−0.019 (2)
C20	0.088 (3)	0.051 (2)	0.068 (2)	−0.009 (2)	−0.025 (2)	−0.0033 (19)
C21	0.052 (2)	0.0349 (17)	0.055 (2)	−0.0025 (15)	−0.0085 (16)	−0.0010 (15)
C22	0.052 (2)	0.0368 (17)	0.052 (2)	−0.0042 (16)	−0.0049 (18)	0.0002 (15)
C23	0.065 (3)	0.081 (3)	0.052 (2)	−0.012 (2)	−0.008 (2)	0.0028 (19)
C24	0.077 (3)	0.096 (3)	0.052 (2)	−0.007 (3)	0.000 (2)	−0.003 (2)
C25	0.063 (3)	0.071 (3)	0.073 (3)	−0.011 (2)	0.004 (2)	−0.003 (2)
C26	0.053 (2)	0.061 (2)	0.081 (3)	−0.0058 (19)	−0.017 (2)	−0.0001 (19)
C27	0.057 (2)	0.050 (2)	0.055 (2)	−0.0056 (18)	−0.0055 (19)	−0.0023 (16)
C28	0.0438 (19)	0.0361 (17)	0.0529 (19)	−0.0090 (15)	−0.0061 (16)	−0.0026 (14)
C29	0.052 (2)	0.0468 (19)	0.053 (2)	−0.0130 (17)	−0.0136 (17)	−0.0023 (16)
C30	0.072 (3)	0.064 (2)	0.072 (3)	−0.009 (2)	−0.030 (2)	−0.001 (2)
C31	0.099 (3)	0.094 (3)	0.085 (3)	−0.014 (3)	−0.049 (3)	0.010 (3)
C32	0.103 (4)	0.103 (3)	0.063 (3)	−0.028 (3)	−0.033 (3)	−0.003 (2)
C33	0.068 (3)	0.071 (2)	0.061 (2)	−0.016 (2)	−0.012 (2)	−0.010 (2)
C34	0.050 (2)	0.052 (2)	0.050 (2)	−0.0125 (17)	−0.0112 (17)	−0.0062 (16)
C35	0.046 (2)	0.0347 (17)	0.055 (2)	−0.0107 (15)	−0.0061 (17)	−0.0043 (16)
C36	0.051 (2)	0.056 (2)	0.072 (2)	−0.0221 (18)	−0.0080 (19)	−0.0040 (18)
C37	0.066 (3)	0.048 (2)	0.069 (2)	−0.0205 (19)	−0.015 (2)	0.0101 (17)
C38	0.065 (2)	0.043 (2)	0.061 (2)	−0.0143 (19)	−0.0249 (19)	−0.0048 (17)
C39	0.0422 (19)	0.0377 (17)	0.0476 (18)	−0.0075 (14)	−0.0096 (15)	−0.0075 (14)
C40	0.050 (2)	0.0497 (19)	0.0470 (18)	−0.0132 (16)	−0.0094 (16)	−0.0059 (15)
C41	0.053 (2)	0.073 (2)	0.062 (2)	−0.0103 (19)	−0.0188 (19)	−0.0040 (19)
C42	0.042 (2)	0.058 (2)	0.071 (2)	−0.0112 (17)	−0.0183 (18)	−0.0025 (18)
C43	0.056 (2)	0.073 (3)	0.081 (3)	−0.014 (2)	−0.024 (2)	−0.007 (2)
C44	0.050 (2)	0.087 (3)	0.103 (3)	−0.029 (2)	−0.012 (2)	−0.001 (3)
C45	0.059 (3)	0.072 (3)	0.093 (3)	−0.010 (2)	0.002 (2)	−0.008 (2)
C46	0.072 (3)	0.082 (3)	0.106 (3)	−0.027 (2)	−0.004 (3)	−0.030 (2)
C47	0.053 (2)	0.076 (3)	0.101 (3)	−0.020 (2)	−0.007 (2)	−0.020 (2)
C48	0.045 (2)	0.0423 (18)	0.057 (2)	−0.0065 (15)	−0.0105 (16)	−0.0114 (15)
C49	0.050 (2)	0.068 (2)	0.066 (2)	−0.0079 (19)	−0.0073 (19)	−0.025 (2)
C50	0.088 (3)	0.100 (3)	0.063 (3)	−0.026 (3)	−0.018 (2)	−0.021 (2)
C51	0.103 (4)	0.173 (6)	0.073 (3)	−0.044 (4)	−0.023 (3)	−0.032 (3)
C52	0.148 (6)	0.164 (7)	0.121 (5)	−0.055 (5)	−0.028 (4)	−0.068 (5)
C53	0.145 (5)	0.112 (5)	0.151 (5)	−0.047 (4)	−0.035 (5)	−0.060 (4)
C54	0.103 (3)	0.072 (3)	0.108 (3)	−0.021 (3)	−0.026 (3)	−0.039 (3)
C55	0.049 (2)	0.0474 (19)	0.0474 (19)	−0.0047 (16)	−0.0108 (16)	−0.0038 (15)
C56	0.049 (2)	0.052 (2)	0.048 (2)	−0.0090 (17)	−0.0048 (17)	−0.0070 (16)
C57	0.064 (3)	0.079 (3)	0.067 (2)	−0.014 (2)	−0.005 (2)	0.005 (2)
C58	0.073 (3)	0.122 (4)	0.067 (3)	−0.028 (3)	0.010 (2)	0.013 (3)
C59	0.054 (3)	0.119 (4)	0.078 (3)	−0.017 (3)	0.002 (2)	−0.003 (3)
C60	0.053 (3)	0.105 (3)	0.073 (3)	−0.007 (2)	−0.009 (2)	0.007 (2)
C61	0.053 (2)	0.078 (3)	0.063 (2)	−0.012 (2)	−0.006 (2)	0.006 (2)
C62	0.0436 (19)	0.0344 (16)	0.0494 (19)	−0.0074 (14)	−0.0102 (16)	−0.0044 (14)
C63	0.048 (2)	0.0404 (18)	0.0499 (19)	−0.0114 (16)	−0.0127 (16)	−0.0029 (15)
C64	0.067 (2)	0.050 (2)	0.068 (2)	−0.0169 (18)	−0.027 (2)	0.0008 (18)
C65	0.097 (3)	0.077 (3)	0.066 (3)	−0.034 (3)	−0.040 (2)	0.014 (2)
C66	0.100 (3)	0.106 (3)	0.047 (2)	−0.046 (3)	−0.021 (2)	0.006 (2)
C67	0.073 (3)	0.075 (3)	0.049 (2)	−0.022 (2)	−0.005 (2)	−0.0110 (19)
C68	0.048 (2)	0.054 (2)	0.047 (2)	−0.0181 (17)	−0.0096 (16)	−0.0044 (16)
C69	0.046 (2)	0.0368 (17)	0.049 (2)	−0.0104 (15)	−0.0097 (17)	−0.0003 (16)
N1	0.0460 (17)	0.0561 (17)	0.0536 (16)	−0.0074 (14)	−0.0061 (14)	−0.0102 (14)
N2	0.0555 (19)	0.0426 (17)	0.073 (2)	−0.0175 (15)	0.0051 (15)	−0.0155 (15)
N3	0.0518 (17)	0.0404 (15)	0.0575 (17)	−0.0031 (13)	−0.0077 (14)	−0.0100 (13)
N4	0.0428 (16)	0.0463 (15)	0.0566 (16)	−0.0145 (13)	−0.0088 (14)	0.0022 (13)
N5	0.0483 (18)	0.0496 (17)	0.0535 (17)	−0.0189 (14)	−0.0016 (13)	−0.0118 (14)
N6	0.0525 (17)	0.0390 (15)	0.0528 (17)	−0.0027 (13)	−0.0072 (14)	−0.0133 (13)
O1	0.0806 (18)	0.0488 (14)	0.0677 (16)	−0.0108 (13)	−0.0125 (13)	0.0071 (12)
O2	0.0708 (16)	0.0410 (13)	0.0555 (14)	−0.0047 (11)	−0.0130 (12)	0.0010 (11)
O3	0.0741 (17)	0.0416 (13)	0.0914 (17)	−0.0062 (13)	−0.0239 (14)	0.0036 (12)
O4	0.0583 (15)	0.0394 (12)	0.0545 (14)	−0.0060 (11)	−0.0097 (12)	−0.0009 (11)

### Geometric parameters (Å, °)

**Table:**

C2—N1	1.453 (4)	C36—H36A	0.97
C2—C3	1.511 (5)	C36—H36B	0.97
C2—H2A	0.97	C37—C38	1.505 (4)
C2—H2B	0.97	C37—H37A	0.97
C3—C4	1.498 (4)	C37—H37B	0.97
C3—H3A	0.97	C38—O3	1.216 (3)
C3—H3B	0.97	C38—C39	1.538 (4)
C4—O1	1.217 (3)	C39—C40	1.531 (4)
C4—C5	1.527 (4)	C39—C48	1.553 (4)
C5—C6	1.531 (4)	C39—C62	1.608 (4)
C5—C14	1.564 (4)	C40—N4	1.459 (3)
C5—C28	1.592 (4)	C40—H40A	0.97
C6—N1	1.459 (3)	C40—H40B	0.97
C6—H6A	0.97	C41—N4	1.467 (4)
C6—H6B	0.97	C41—C42	1.504 (4)
C7—N1	1.469 (4)	C41—H41A	0.97
C7—C8	1.504 (4)	C41—H41B	0.97
C7—H7A	0.97	C42—C47	1.371 (4)
C7—H7B	0.97	C42—C43	1.380 (4)
C8—C9	1.356 (4)	C43—C44	1.386 (5)
C8—C13	1.362 (5)	C43—H43	0.93
C9—C10	1.369 (5)	C44—C45	1.357 (5)
C9—H9	0.93	C44—H44	0.93
C10—C11	1.344 (5)	C45—C46	1.369 (5)
C10—H10	0.93	C45—H45	0.93
C11—C12	1.334 (6)	C46—C47	1.381 (5)
C11—H11	0.93	C46—H46	0.93
C12—C13	1.363 (6)	C47—H47	0.93
C12—H12	0.93	C48—C49	1.505 (4)
C13—H13	0.93	C48—C55	1.536 (4)
C14—C15	1.516 (4)	C48—H48	0.98
C14—C21	1.536 (4)	C49—C54	1.377 (5)
C14—H14	0.98	C49—C50	1.389 (5)
C15—C16	1.381 (4)	C50—C51	1.384 (5)
C15—C20	1.394 (4)	C50—H50	0.93
C16—C17	1.379 (5)	C51—C52	1.353 (7)
C16—H16	0.93	C51—H51	0.93
C17—C18	1.375 (5)	C52—C53	1.379 (8)
C17—H17	0.93	C52—H52	0.93
C18—C19	1.368 (5)	C53—C54	1.411 (6)
C18—H18	0.93	C53—H53	0.93
C19—C20	1.377 (5)	C54—H54	0.93
C19—H19	0.93	C55—N5	1.471 (4)
C20—H20	0.93	C55—C56	1.501 (4)
C21—N2	1.463 (4)	C55—H55	0.98
C21—C22	1.514 (4)	C56—C61	1.373 (4)
C21—H21	0.98	C56—C57	1.377 (4)
C22—C27	1.373 (4)	C57—C58	1.374 (5)
C22—C23	1.380 (4)	C57—H57	0.93
C23—C24	1.384 (5)	C58—C59	1.368 (5)
C23—H23	0.93	C58—H58	0.93
C24—C25	1.350 (5)	C59—C60	1.352 (5)
C24—H24	0.93	C59—H59	0.93
C25—C26	1.375 (5)	C60—C61	1.373 (5)
C25—H25	0.93	C60—H60	0.93
C26—C27	1.385 (4)	C61—H61	0.93
C26—H26	0.93	C62—N5	1.463 (4)
C27—H27	0.93	C62—C63	1.509 (4)
C28—N2	1.461 (4)	C62—C69	1.558 (4)
C28—C29	1.509 (4)	C63—C64	1.375 (4)
C28—C35	1.553 (4)	C63—C68	1.386 (4)
C29—C30	1.366 (4)	C64—C65	1.390 (4)
C29—C34	1.394 (4)	C64—H64	0.93
C30—C31	1.379 (5)	C65—C66	1.374 (5)
C30—H30	0.93	C65—H65	0.93
C31—C32	1.380 (5)	C66—C67	1.375 (5)
C31—H31	0.93	C66—H66	0.93
C32—C33	1.382 (5)	C67—C68	1.369 (4)
C32—H32	0.93	C67—H67	0.93
C33—C34	1.368 (4)	C68—N6	1.399 (4)
C33—H33	0.93	C69—O4	1.232 (3)
C34—N3	1.399 (4)	C69—N6	1.343 (3)
C35—O2	1.229 (3)	N2—H2	0.817 (17)
C35—N3	1.351 (4)	N3—H3	0.86
C36—N4	1.453 (4)	N5—H5	0.835 (18)
C36—C37	1.510 (4)	N6—H6	0.86
			
N1—C2—C3	110.5 (3)	C38—C37—H37B	108.9
N1—C2—H2A	109.5	C36—C37—H37B	108.9
C3—C2—H2A	109.5	H37A—C37—H37B	107.7
N1—C2—H2B	109.5	O3—C38—C37	121.8 (3)
C3—C2—H2B	109.5	O3—C38—C39	122.2 (3)
H2A—C2—H2B	108.1	C37—C38—C39	116.0 (3)
C4—C3—C2	112.6 (3)	C40—C39—C38	106.9 (2)
C4—C3—H3A	109.1	C40—C39—C48	112.3 (2)
C2—C3—H3A	109.1	C38—C39—C48	111.9 (2)
C4—C3—H3B	109.1	C40—C39—C62	112.8 (2)
C2—C3—H3B	109.1	C38—C39—C62	110.0 (2)
H3A—C3—H3B	107.8	C48—C39—C62	103.1 (2)
O1—C4—C3	121.6 (3)	N4—C40—C39	110.9 (2)
O1—C4—C5	121.8 (3)	N4—C40—H40A	109.5
C3—C4—C5	116.6 (3)	C39—C40—H40A	109.5
C4—C5—C6	106.4 (3)	N4—C40—H40B	109.5
C4—C5—C14	111.8 (2)	C39—C40—H40B	109.5
C6—C5—C14	112.0 (2)	H40A—C40—H40B	108.0
C4—C5—C28	111.2 (2)	N4—C41—C42	111.7 (3)
C6—C5—C28	112.6 (2)	N4—C41—H41A	109.3
C14—C5—C28	103.1 (2)	C42—C41—H41A	109.3
N1—C6—C5	111.9 (2)	N4—C41—H41B	109.3
N1—C6—H6A	109.2	C42—C41—H41B	109.3
C5—C6—H6A	109.2	H41A—C41—H41B	107.9
N1—C6—H6B	109.2	C47—C42—C43	118.1 (3)
C5—C6—H6B	109.2	C47—C42—C41	120.4 (3)
H6A—C6—H6B	107.9	C43—C42—C41	121.5 (3)
N1—C7—C8	113.1 (3)	C42—C43—C44	120.6 (3)
N1—C7—H7A	109.0	C42—C43—H43	119.7
C8—C7—H7A	109.0	C44—C43—H43	119.7
N1—C7—H7B	109.0	C45—C44—C43	120.5 (4)
C8—C7—H7B	109.0	C45—C44—H44	119.7
H7A—C7—H7B	107.8	C43—C44—H44	119.7
C9—C8—C13	117.3 (3)	C44—C45—C46	119.4 (4)
C9—C8—C7	120.6 (3)	C44—C45—H45	120.3
C13—C8—C7	122.1 (3)	C46—C45—H45	120.3
C8—C9—C10	121.2 (4)	C45—C46—C47	120.3 (4)
C8—C9—H9	119.4	C45—C46—H46	119.9
C10—C9—H9	119.4	C47—C46—H46	119.9
C11—C10—C9	120.5 (4)	C42—C47—C46	121.0 (4)
C11—C10—H10	119.7	C42—C47—H47	119.5
C9—C10—H10	119.7	C46—C47—H47	119.5
C12—C11—C10	118.8 (4)	C49—C48—C55	116.2 (3)
C12—C11—H11	120.6	C49—C48—C39	116.1 (3)
C10—C11—H11	120.6	C55—C48—C39	103.6 (2)
C11—C12—C13	121.4 (4)	C49—C48—H48	106.7
C11—C12—H12	119.3	C55—C48—H48	106.7
C13—C12—H12	119.3	C39—C48—H48	106.7
C8—C13—C12	120.8 (4)	C54—C49—C50	118.8 (4)
C8—C13—H13	119.6	C54—C49—C48	118.7 (4)
C12—C13—H13	119.6	C50—C49—C48	122.5 (3)
C15—C14—C21	116.4 (3)	C51—C50—C49	121.2 (5)
C15—C14—C5	115.6 (3)	C51—C50—H50	119.4
C21—C14—C5	103.8 (2)	C49—C50—H50	119.4
C15—C14—H14	106.8	C52—C51—C50	119.9 (6)
C21—C14—H14	106.8	C52—C51—H51	120.0
C5—C14—H14	106.8	C50—C51—H51	120.0
C16—C15—C20	116.5 (3)	C51—C52—C53	120.6 (6)
C16—C15—C14	120.1 (3)	C51—C52—H52	119.7
C20—C15—C14	123.4 (3)	C53—C52—H52	119.7
C17—C16—C15	121.9 (4)	C52—C53—C54	119.7 (6)
C17—C16—H16	119.1	C52—C53—H53	120.2
C15—C16—H16	119.1	C54—C53—H53	120.2
C18—C17—C16	120.3 (4)	C49—C54—C53	119.8 (5)
C18—C17—H17	119.9	C49—C54—H54	120.1
C16—C17—H17	119.9	C53—C54—H54	120.1
C19—C18—C17	119.2 (4)	N5—C55—C56	111.2 (3)
C19—C18—H18	120.4	N5—C55—C48	100.3 (2)
C17—C18—H18	120.4	C56—C55—C48	115.5 (2)
C18—C19—C20	120.2 (4)	N5—C55—H55	109.8
C18—C19—H19	119.9	C56—C55—H55	109.8
C20—C19—H19	119.9	C48—C55—H55	109.8
C19—C20—C15	121.9 (3)	C61—C56—C57	117.4 (3)
C19—C20—H20	119.1	C61—C56—C55	121.5 (3)
C15—C20—H20	119.1	C57—C56—C55	121.1 (3)
N2—C21—C22	111.6 (3)	C58—C57—C56	121.1 (4)
N2—C21—C14	100.5 (2)	C58—C57—H57	119.5
C22—C21—C14	114.2 (2)	C56—C57—H57	119.5
N2—C21—H21	110.0	C59—C58—C57	120.0 (4)
C22—C21—H21	110.0	C59—C58—H58	120.0
C14—C21—H21	110.0	C57—C58—H58	120.0
C27—C22—C23	118.3 (3)	C60—C59—C58	119.9 (4)
C27—C22—C21	120.7 (3)	C60—C59—H59	120.1
C23—C22—C21	120.9 (3)	C58—C59—H59	120.1
C22—C23—C24	120.9 (4)	C59—C60—C61	119.9 (4)
C22—C23—H23	119.6	C59—C60—H60	120.0
C24—C23—H23	119.6	C61—C60—H60	120.0
C25—C24—C23	120.2 (4)	C60—C61—C56	121.7 (3)
C25—C24—H24	119.9	C60—C61—H61	119.1
C23—C24—H24	119.9	C56—C61—H61	119.1
C24—C25—C26	120.0 (4)	N5—C62—C63	110.5 (2)
C24—C25—H25	120.0	N5—C62—C69	110.6 (2)
C26—C25—H25	120.0	C63—C62—C69	100.8 (2)
C25—C26—C27	119.9 (4)	N5—C62—C39	103.8 (2)
C25—C26—H26	120.1	C63—C62—C39	118.1 (2)
C27—C26—H26	120.1	C69—C62—C39	113.2 (2)
C22—C27—C26	120.7 (3)	C64—C63—C68	119.5 (3)
C22—C27—H27	119.6	C64—C63—C62	131.1 (3)
C26—C27—H27	119.6	C68—C63—C62	109.3 (3)
N2—C28—C29	111.4 (3)	C63—C64—C65	119.3 (3)
N2—C28—C35	111.1 (2)	C63—C64—H64	120.3
C29—C28—C35	101.3 (2)	C65—C64—H64	120.3
N2—C28—C5	103.7 (2)	C66—C65—C64	119.5 (4)
C29—C28—C5	115.4 (2)	C66—C65—H65	120.2
C35—C28—C5	114.1 (2)	C64—C65—H65	120.2
C30—C29—C34	119.5 (3)	C65—C66—C67	121.9 (3)
C30—C29—C28	131.6 (3)	C65—C66—H66	119.0
C34—C29—C28	108.9 (3)	C67—C66—H66	119.0
C29—C30—C31	119.2 (4)	C68—C67—C66	117.8 (3)
C29—C30—H30	120.4	C68—C67—H67	121.1
C31—C30—H30	120.4	C66—C67—H67	121.1
C30—C31—C32	120.3 (4)	C67—C68—C63	121.8 (3)
C30—C31—H31	119.8	C67—C68—N6	128.6 (3)
C32—C31—H31	119.8	C63—C68—N6	109.6 (3)
C31—C32—C33	121.6 (4)	O4—C69—N6	125.5 (3)
C31—C32—H32	119.2	O4—C69—C62	125.7 (3)
C33—C32—H32	119.2	N6—C69—C62	108.5 (3)
C34—C33—C32	116.9 (3)	C2—N1—C6	109.0 (3)
C34—C33—H33	121.5	C2—N1—C7	112.3 (3)
C32—C33—H33	121.5	C6—N1—C7	108.9 (2)
C33—C34—C29	122.5 (3)	C28—N2—C21	112.3 (3)
C33—C34—N3	128.1 (3)	C28—N2—H2	117 (2)
C29—C34—N3	109.4 (3)	C21—N2—H2	113 (2)
O2—C35—N3	125.7 (3)	C35—N3—C34	112.0 (3)
O2—C35—C28	126.1 (3)	C35—N3—H3	124.0
N3—C35—C28	108.0 (3)	C34—N3—H3	124.0
N4—C36—C37	110.3 (3)	C36—N4—C40	108.6 (2)
N4—C36—H36A	109.6	C36—N4—C41	111.3 (3)
C37—C36—H36A	109.6	C40—N4—C41	111.3 (2)
N4—C36—H36B	109.6	C62—N5—C55	108.4 (2)
C37—C36—H36B	109.6	C62—N5—H5	115 (3)
H36A—C36—H36B	108.1	C55—N5—H5	110 (3)
C38—C37—C36	113.3 (3)	C69—N6—C68	111.8 (2)
C38—C37—H37A	108.9	C69—N6—H6	124.1
C36—C37—H37A	108.9	C68—N6—H6	124.1
			
N1—C2—C3—C4	50.3 (4)	C41—C42—C43—C44	179.7 (3)
C2—C3—C4—O1	134.1 (3)	C42—C43—C44—C45	−0.5 (6)
C2—C3—C4—C5	−44.1 (4)	C43—C44—C45—C46	−0.7 (6)
O1—C4—C5—C6	−132.6 (3)	C44—C45—C46—C47	1.4 (7)
C3—C4—C5—C6	45.6 (3)	C43—C42—C47—C46	−0.4 (6)
O1—C4—C5—C14	−10.1 (4)	C41—C42—C47—C46	−179.1 (4)
C3—C4—C5—C14	168.1 (3)	C45—C46—C47—C42	−0.8 (7)
O1—C4—C5—C28	104.5 (3)	C40—C39—C48—C49	−32.7 (4)
C3—C4—C5—C28	−77.3 (3)	C38—C39—C48—C49	87.5 (3)
C4—C5—C6—N1	−57.0 (3)	C62—C39—C48—C49	−154.3 (3)
C14—C5—C6—N1	−179.4 (2)	C40—C39—C48—C55	96.1 (3)
C28—C5—C6—N1	65.0 (3)	C38—C39—C48—C55	−143.8 (2)
N1—C7—C8—C9	−58.4 (5)	C62—C39—C48—C55	−25.6 (3)
N1—C7—C8—C13	123.3 (4)	C55—C48—C49—C54	149.9 (3)
C13—C8—C9—C10	0.1 (6)	C39—C48—C49—C54	−87.8 (4)
C7—C8—C9—C10	−178.3 (3)	C55—C48—C49—C50	−28.8 (5)
C8—C9—C10—C11	0.6 (6)	C39—C48—C49—C50	93.5 (4)
C9—C10—C11—C12	−0.6 (7)	C54—C49—C50—C51	1.2 (6)
C10—C11—C12—C13	0.0 (8)	C48—C49—C50—C51	179.9 (4)
C9—C8—C13—C12	−0.8 (7)	C49—C50—C51—C52	0.5 (7)
C7—C8—C13—C12	177.6 (5)	C50—C51—C52—C53	−1.9 (9)
C11—C12—C13—C8	0.8 (9)	C51—C52—C53—C54	1.4 (10)
C4—C5—C14—C15	−81.1 (3)	C50—C49—C54—C53	−1.7 (6)
C6—C5—C14—C15	38.1 (4)	C48—C49—C54—C53	179.6 (4)
C28—C5—C14—C15	159.4 (3)	C52—C53—C54—C49	0.4 (8)
C4—C5—C14—C21	150.1 (3)	C49—C48—C55—N5	171.2 (3)
C6—C5—C14—C21	−90.7 (3)	C39—C48—C55—N5	42.5 (3)
C28—C5—C14—C21	30.6 (3)	C49—C48—C55—C56	−69.2 (4)
C21—C14—C15—C16	−142.3 (3)	C39—C48—C55—C56	162.1 (3)
C5—C14—C15—C16	95.4 (3)	N5—C55—C56—C61	53.0 (4)
C21—C14—C15—C20	39.0 (4)	C48—C55—C56—C61	−60.4 (4)
C5—C14—C15—C20	−83.3 (4)	N5—C55—C56—C57	−126.0 (3)
C20—C15—C16—C17	2.0 (5)	C48—C55—C56—C57	120.6 (3)
C14—C15—C16—C17	−176.8 (3)	C61—C56—C57—C58	1.0 (5)
C15—C16—C17—C18	−0.1 (5)	C55—C56—C57—C58	−179.9 (3)
C16—C17—C18—C19	−1.7 (6)	C56—C57—C58—C59	−0.4 (6)
C17—C18—C19—C20	1.4 (6)	C57—C58—C59—C60	−0.5 (7)
C18—C19—C20—C15	0.6 (6)	C58—C59—C60—C61	0.8 (7)
C16—C15—C20—C19	−2.3 (5)	C59—C60—C61—C56	−0.1 (6)
C14—C15—C20—C19	176.4 (3)	C57—C56—C61—C60	−0.7 (5)
C15—C14—C21—N2	−168.4 (3)	C55—C56—C61—C60	−179.8 (3)
C5—C14—C21—N2	−40.2 (3)	C40—C39—C62—N5	−122.1 (3)
C15—C14—C21—C22	71.9 (4)	C38—C39—C62—N5	118.7 (3)
C5—C14—C21—C22	−159.8 (3)	C48—C39—C62—N5	−0.8 (3)
N2—C21—C22—C27	−36.7 (4)	C40—C39—C62—C63	115.3 (3)
C14—C21—C22—C27	76.5 (4)	C38—C39—C62—C63	−3.9 (4)
N2—C21—C22—C23	144.9 (3)	C48—C39—C62—C63	−123.4 (3)
C14—C21—C22—C23	−102.0 (4)	C40—C39—C62—C69	−2.1 (3)
C27—C22—C23—C24	0.4 (5)	C38—C39—C62—C69	−121.3 (3)
C21—C22—C23—C24	178.9 (3)	C48—C39—C62—C69	119.1 (3)
C22—C23—C24—C25	0.1 (6)	N5—C62—C63—C64	−59.3 (4)
C23—C24—C25—C26	−0.5 (6)	C69—C62—C63—C64	−176.2 (3)
C24—C25—C26—C27	0.3 (6)	C39—C62—C63—C64	59.9 (4)
C23—C22—C27—C26	−0.6 (5)	N5—C62—C63—C68	117.7 (3)
C21—C22—C27—C26	−179.1 (3)	C69—C62—C63—C68	0.7 (3)
C25—C26—C27—C22	0.2 (5)	C39—C62—C63—C68	−123.2 (3)
C4—C5—C28—N2	−129.1 (3)	C68—C63—C64—C65	3.4 (5)
C6—C5—C28—N2	111.6 (3)	C62—C63—C64—C65	−179.9 (3)
C14—C5—C28—N2	−9.2 (3)	C63—C64—C65—C66	−1.5 (5)
C4—C5—C28—C29	−6.9 (4)	C64—C65—C66—C67	−1.1 (6)
C6—C5—C28—C29	−126.2 (3)	C65—C66—C67—C68	1.6 (6)
C14—C5—C28—C29	113.0 (3)	C66—C67—C68—C63	0.4 (5)
C4—C5—C28—C35	109.9 (3)	C66—C67—C68—N6	−178.8 (3)
C6—C5—C28—C35	−9.4 (3)	C64—C63—C68—C67	−2.9 (5)
C14—C5—C28—C35	−130.3 (3)	C62—C63—C68—C67	179.7 (3)
N2—C28—C29—C30	55.0 (4)	C64—C63—C68—N6	176.4 (3)
C35—C28—C29—C30	173.3 (3)	C62—C63—C68—N6	−1.0 (3)
C5—C28—C29—C30	−62.9 (4)	N5—C62—C69—O4	56.2 (4)
N2—C28—C29—C34	−123.5 (3)	C63—C62—C69—O4	173.1 (3)
C35—C28—C29—C34	−5.3 (3)	C39—C62—C69—O4	−59.7 (4)
C5—C28—C29—C34	118.5 (3)	N5—C62—C69—N6	−117.0 (3)
C34—C29—C30—C31	−1.4 (5)	C63—C62—C69—N6	−0.2 (3)
C28—C29—C30—C31	−179.8 (4)	C39—C62—C69—N6	127.0 (3)
C29—C30—C31—C32	1.0 (6)	C3—C2—N1—C6	−62.1 (3)
C30—C31—C32—C33	−0.6 (7)	C3—C2—N1—C7	177.2 (3)
C31—C32—C33—C34	0.4 (6)	C5—C6—N1—C2	68.0 (3)
C32—C33—C34—C29	−0.8 (5)	C5—C6—N1—C7	−169.2 (3)
C32—C33—C34—N3	175.9 (3)	C8—C7—N1—C2	−61.0 (4)
C30—C29—C34—C33	1.3 (5)	C8—C7—N1—C6	178.3 (3)
C28—C29—C34—C33	−179.9 (3)	C29—C28—N2—C21	−142.1 (3)
C30—C29—C34—N3	−175.9 (3)	C35—C28—N2—C21	105.7 (3)
C28—C29—C34—N3	2.8 (3)	C5—C28—N2—C21	−17.3 (3)
N2—C28—C35—O2	−49.5 (4)	C22—C21—N2—C28	158.1 (3)
C29—C28—C35—O2	−168.0 (3)	C14—C21—N2—C28	36.6 (3)
C5—C28—C35—O2	67.3 (4)	O2—C35—N3—C34	169.2 (3)
N2—C28—C35—N3	124.5 (3)	C28—C35—N3—C34	−4.8 (3)
C29—C28—C35—N3	6.0 (3)	C33—C34—N3—C35	−175.6 (3)
C5—C28—C35—N3	−118.7 (3)	C29—C34—N3—C35	1.4 (4)
N4—C36—C37—C38	−49.7 (4)	C37—C36—N4—C40	63.3 (3)
C36—C37—C38—O3	−137.5 (3)	C37—C36—N4—C41	−173.8 (3)
C36—C37—C38—C39	42.0 (4)	C39—C40—N4—C36	−69.9 (3)
O3—C38—C39—C40	135.0 (3)	C39—C40—N4—C41	167.3 (2)
C37—C38—C39—C40	−44.5 (3)	C42—C41—N4—C36	61.8 (3)
O3—C38—C39—C48	11.7 (4)	C42—C41—N4—C40	−176.9 (3)
C37—C38—C39—C48	−167.8 (3)	C63—C62—N5—C55	156.4 (2)
O3—C38—C39—C62	−102.3 (3)	C69—C62—N5—C55	−92.8 (3)
C37—C38—C39—C62	78.2 (3)	C39—C62—N5—C55	28.9 (3)
C38—C39—C40—N4	57.9 (3)	C56—C55—N5—C62	−167.9 (3)
C48—C39—C40—N4	−179.0 (2)	C48—C55—N5—C62	−45.3 (3)
C62—C39—C40—N4	−63.1 (3)	O4—C69—N6—C68	−173.7 (3)
N4—C41—C42—C47	64.6 (4)	C62—C69—N6—C68	−0.4 (3)
N4—C41—C42—C43	−114.0 (4)	C67—C68—N6—C69	−179.9 (3)
C47—C42—C43—C44	1.1 (5)	C63—C68—N6—C69	0.9 (3)

### Hydrogen-bond geometry (Å, °)

**Table:**

*D*—H···*A*	*D*—H	H···*A*	*D*···*A*	*D*—H···*A*
N3—H3···O4	0.86	2.03	2.881 (3)	169
N6—H6···O2	0.86	2.05	2.896 (3)	170
C40—H40A···O4	0.97	2.35	2.925 (4)	118
C12—H12···Cg1^i^	0.93	2.80	3.586 (6)	144

Symmetry codes: (i) *x*+1, *y*, *z*.
